# BEFORE A NEUROCOGNITIVE EVALUATION: ROLES AND COMPETENCIES OF BEHAVIORAL HEALTH PROVIDERS

**DOI:** 10.1093/geroni/igac059.793

**Published:** 2022-12-20

**Authors:** Ann Steffen, Rebecca Allen

**Affiliations:** University of Missouri-St. Louis, St. Louis, Missouri, United States; The University of Alabama, Tuscaloosa, Alabama, United States

## Abstract

Neurocognitive disorders are among the most stigmatized medical conditions in the USA and across the globe. Cultural beliefs about cognitive aging also impact how individuals, families and healthcare teams respond to concerns about cognitive impairment. GSA’s revised KAER Toolkit for Primary Care Teams (Fall, 2020) provides useful information for the early stages of this process, including kickstarting the conversation about brain health concerns and conducting screenings. This presentation outlines additional competencies for behavioral health clinicians involved in patient, family, and health care team discussions regarding cognitive health concerns. Culturally responsive strategies and resources are suggested to help clinicians explore the decision for a formal evaluation and shape the nature of the referral question(s).

